# Ligustilide attenuates nitric oxide‐induced apoptosis in rat chondrocytes and cartilage degradation via inhibiting JNK and p38 MAPK pathways

**DOI:** 10.1111/jcmm.14226

**Published:** 2019-02-15

**Authors:** Yan Zhou, Jianghua Ming, Yaming Li, Ming Deng, Qing Chen, Yonggang Ma, Zhonghui Chen, Yubiao Zhang, Shiqing Liu

**Affiliations:** ^1^ Department of Orthopedics Central Laboratory Renmin Hospital of Wuhan University Wuhan China

**Keywords:** apoptosis, JNK, ligustilide, osteoarthritis, p38 MAPK

## Abstract

Ligustilide (LIG) is the main lipophilic component of the Umbelliferae family of pharmaceutical plants, including Radix angelicae sinensis and Ligusticum chuanxiong. LIG shows various pharmacological properties associated with anti‐inflammation and anti‐apoptosis in several kinds of cell lines. However, the therapeutic effects of LIG on chondrocyte apoptosis remain unknown. In this study, we investigated whether LIG had an anti‐apoptotic activity in sodium nitroprusside (SNP)‐stimulated chondrocyte apoptosis and could delay cartilage degeneration in a surgically induced rat OA model, and elucidated the potential mechanisms. In vitro studies revealed that LIG significantly suppressed chondrocyte apoptosis and cytoskeletal remodelling, which maintained the nuclear morphology and increased the mitochondrial membrane potential. In terms of SNP, LIG treatment considerably reduced the expression levels of cleaved caspase‐3, Bax and inducible nitric oxide synthase and increased the expression level of Bcl‐2 in a dose‐dependent manner. The LIG‐treated groups presented a significantly suppressed expression of activating transcription factor 2 and phosphorylation of Jun N‐terminal kinase (JNK) and p38 mitogen‐activated protein kinase (MAPK). The inhibitory effect of LIG was enhanced by the p38 MAPK inhibitor SB203580 or the JNK inhibitor SP600125 and offset by the agonist anisomycin. In vivo studies demonstrated that LIG attenuated osteoarthritic cartilage destruction by inhibiting the cartilage chondrocyte apoptosis and suppressing the phosphorylation levels of activating transcription factor 2, JNK and p38 MAPK. This result was confirmed by histological analyses, micro‐CT, TUNEL assay and immunohistochemical analyses. Collectively, our studies indicated that LIG protected chondrocytes against SNP‐induced apoptosis and delayed articular cartilage degeneration by suppressing JNK and p38 MAPK pathways.

## 1 INTRODUCTION

Osteoarthritis (OA) is a joint disorder disease associated with synovial inflammation, articular cartilage degradation, subchondral bone remodelling and osteophyte formation. These pathological events result in severe consequences, including chronic pain, functional disability and destruction of joint architectural integrity.^1^, ^2^ During the initiation and development of OA, chondrocyte apoptosis and inflammation play critical roles in OA cartilage degradation.^3^

Chondrocyte apoptosis is a major factor contributing to cartilage degeneration and possible target for OA treatment.^4^ The activation of caspase‐3 is vital in apoptosis because it facilitates the hydrolysis of cytoskeletal proteins and nucleic acids. The growth of caspase‐3 in chondrocytes can be promoted by nitric oxide (NO), and caspase‐3 activity is mainly stimulated by cytochrome c (Cyt c).^5^ Bcl‐2 family members, containing Bcl‐2 and Bax, can trigger Cyt c release from the mitochondria and regulate caspase‐3 activity signalling processes. The main approach against apoptosis is to prevent caspase‐3 activation, which can be decreased by Bcl‐2 through binding to and hindering Bax throughout the mitochondrion‐related pathway.^6^

Mitogen‐activated protein kinase (MAPK), a member of the intracellular serine‐threonine protein kinase superfamily, is a vital part of multiple signal transduction pathways. In general, the MAPK pathway involves three signal cascades: c‐Jun N‐terminal kinase (JNK), p38 and extracellular signal‐regulated kinase.^7^ As two major pathways of MAPK signal transduction, JNK and p38 play important roles in the stimulation of apoptotic signalling and inflammatory diseases, especially OA.^8^ Many downstream targets in the JNK and p38 signal pathways include various sets of transcription elements through which they can activate complex changes to gene expression. In the activator protein 1 family of bZip‐containing transcription factors, activating transcription factor 2 (ATF2) is a substrate commonly activated by the stress‐activated protein kinases p38 and JNK. Reacting to various stimuli, these kinases also phosphorylate ATF2 at the two main threonine residues in the N‐terminal transactivation domain, resulting in its activation.^9^ In the Kashin‐Beck disease, the activation and function of ATF2 by JNK and p38 signalling pathways participate in cartilage chondrocyte apoptosis.^10^

**Figure 1:**
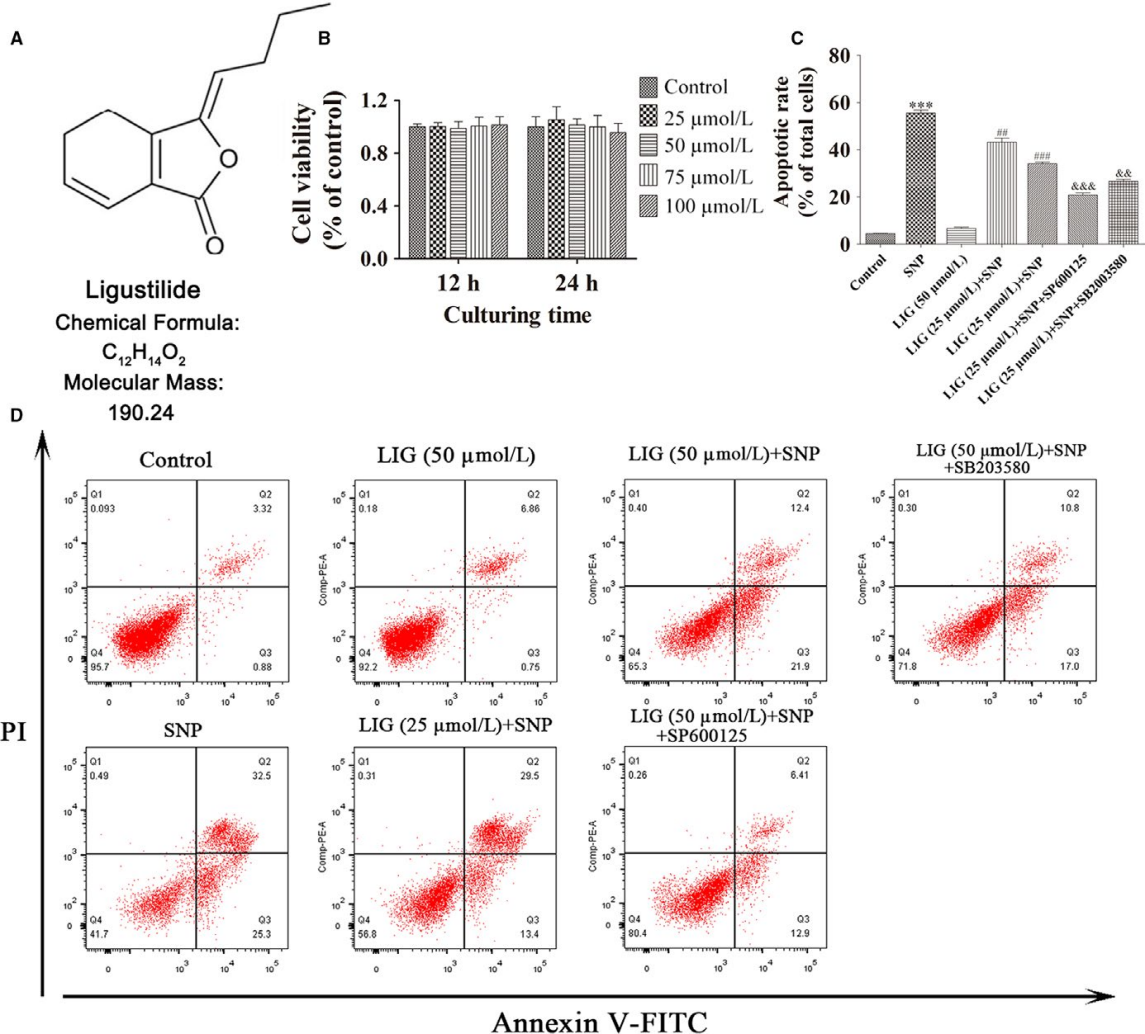
Treatment with ligustilide (LIG) protects chondrocytes from sodium nitroprusside (SNP)‐induced apoptosis. A, Chemical structure of LIG. B, Chondrocytes were treated with LIG in a dose‐dependent manner. After 12 and 24 h of culture, cell viability was evaluated using the Cell counting kit‐8 (CCK‐8) assay. C, D, Chondrocytes treated with SNP (0.75 mmol/L) were untreated or co‐treated with or without LIG (25 and 50 μmol/L) and the JNK inhibitor SP600125 (10 μmol/L) or the p38 mitogen‐activated protein kinase inhibitor SB203580 (10 μmol/L) for 24 h. The chondrocyte apoptotic rate was assessed through flow cytometry with Annexin V‐fluorescein isothiocyanate (FITC)/propidium iodide (PI) dual staining. Each column represented mean ± SEM in the CCK‐8 assay (n = 6) and in flow cytometry (n = 3). ***P < 0.01 vs the control group. ^##^ *P *< 0.01 and ^###^ *P *< 0.001 vs the SNP group; ^&&^ *P *< 0.01 and ^&&&^ *P *< 0.001 vs the SNP + LIG (50 μmol/L) group

Ligustilide (LIG; structure shown in Figure 1A) is the main lipophilic component of the Umbelliferae family, including Radix angelicae sinensis and Ligusticum chuanxiong.^11^ LIG shows various pharmacological effects, including neuroprotective,^12^ vasodilatating,^13^ antitumor,^14^ anti‐apoptotic,^15^ anti‐inflammatory,^16^ and antioxidant effects.^17^ LIG, as an effective chemotherapeutic agent, attenuates IL‐1β‐induced inflammation and promotes extracellular matrix synthesis in chondrocytes by suppressing NF‐κB activation via the PI3K/AKT pathway.^18^ Nevertheless, the therapeutic mechanism of LIG in chondrocyte apoptosis has yet to be reported. In the current study, we explored whether LIG prevented rat chondrocyte apoptosis and delayed the recessive process of cartilage degeneration. We also investigated the potential molecular mechanisms of the anti‐apoptotic effect of LIG on SNP‐stimulated rat chondrocytes by regulating the JNK and p38 MAPK pathways.

## 2 MATERIALS AND METHODS

### 2.1 Reagents

Ligustilide (LIG) (purity ≥ 98%) was procured from Sigma‐Aldrich (SMB00400, St. Louis, MO, USA). LIG was dissolved in DMSO for stock preparation, and the concentration of 0.05% DMSO was used as the control. The primary antibodies for cleaved caspase‐3 (#9664), Bax (#2772), p‐JNK (#9255) and JNK (#9252) were procured from Cell Signaling Technology (Boston, MA, USA). The antibodies for Bcl‐2 (ab196495), inducible nitric oxide synthase (iNOS) (ab3523), ATF2 (ab47476) and glyceraldehyde‐3‐phosphate dehydrogenase (GAPDH) (ab37168) were procured from Abcam (Cambridge, UK). The antibodies for phospho‐p38 MAPK (p‐p38 MAPK) (AF4001) and p38 MAPK (AF6456) were procured from Affinity Biosciences (Cincinnati, OH, USA). The primary antibody for p‐ATF2 was procured from Bioss (bs‐8449R, Beijing, China). SP600125 (S1460) and anisomycin (S7409) were procured from Selleck Chemicals (Houston, TX, USA). SB203580 was procured from MedChemExpress (HY‐10256, Monmouth, NJ, USA). The vimentin antibody was supplied by Santa Cruz Biotech (sc‐6260, Dallas, USA). Cell counting kit‐8 (CCK‐8) was purchased from Dojindo Laboratories (CK04, Kumamoto, Japan). TUNEL kit was supplied by KeyGEN Biotech (KGA‐701_TY1163, Nanjing, China). The apoptosis kit of annexin V‐fluorescein isothiocyanate (FITC)/propidium iodide (PI) was purchased from MultiSciences Biotech Co., Ltd (70‐AP101‐100, Hangzhou, China). SNP was supplied by Youcare Pharmaceutical Group Co., Ltd (1008, Beijing, China). Dulbecco's modified Eagle's medium (DMEM)/F12 was procured from Hyclone (USA).

### 2.2 Cell culture

Rat chondrocytes derived from the knee joint cartilage of newborn Sprague‐Dawley (SD) rats were purchased from the Center for Animal Experiment/ABSL‐III Laboratory of Wuhan University, Wuhan, China. All protocols were carried out in accordance with the Institutional Ethics Committee of Medical School, Wuhan University. The rat cartilage pieces were digested with 0.25% trypsin and transferred to 0.2% collagenase type II for 4 hours at 37°C. After collection of cells by centrifugation, individual chondrocytes were suspended in DMEM/F12 complete culture medium (containing 100 units/mL of penicillin and streptomycin and 10% FBS) and incubated in a humidified 5% CO2 at 37°C.

### 2.3 Cell viability assay

The chondrocyte viability was assessed in the presence of different concentrations of LIG (0, 25, 50, 70 and 100 μmol/L) using the CCK‐8 assay. For quantitative analysis, 10 μL CCK‐8 solution was added to 100 μL medium and incubated at 37°C for 4 hours. Then, the optical densities of the cells at 450 nm were measured with a microplate reader (Bio‐Tek, Model EXL800, Winooski, VT, USA). Cell viability was calculated as a proportion of the control group.

### 2.4 Flow cytometric evaluation

Cell apoptosis rate was detected by flow cytometry according to the manufacturer's protocol. Briefly, cells and cultural supernatants were collected and centrifuged for 5 minutes at 1000 r.p.m, and washed twice with cold PBS. The cells were smoothly resuspended in 500 μL of binding buffer. Then, PI solution (5 μL) and FITC‐labelled Annexin V (5 μL) were incubated with cells in the dark for 15 minutes. The ratio of cell apoptosis was detected using a FACScan flow cytometer (Becton Dickinson, Franklin Lakes, NJ, USA).

### 2.5 Hoechst 33342 nuclear staining

After each treatment, the cells were fixed in 4% (v/v) paraformaldehyde (GuGe Biotechnology, Wuhan, China) for 30 minutes, followed by 5 μg/mL Hoechst 33342 dye (Beyotime Institute of Biotechnology, Haimen, China) for 20 minutes. Finally, images were taken using an Olympus microscope (Olympus Corporation, Tokyo, Japan). The apoptotic cell ratio was calculated by the proportion of the cells with nucleic morphological changes to the total cells in five random visual areas.

### 2.6 Mitochondrial membrane potential analysis

Rhodamine‐123 fluorescent dye (Sigma‐Aldrich, St. Louis, MO, USA) was employed to detect mitochondrial membrane potential. The culture medium containing different concentrations of LIG was added to treat for 2 hours, followed by SNP (0.75 mmol/L) co‐treatment for 24 hours. After each treatment, the cells were incubated with 2 mL of DMEM/F12 complete culture medium with 10 μmol/L Rhodamine‐123 for 30 minutes at 37°C. Then, the cells were rinsed twice with PBS, and fluorescence signals were imagined under an Olympus microscope. The mitochondrial membrane potential was measured using Image‐Pro Plus 6.0 software.

### 2.7 Immunofluorescence assay

After each treatment, the cells were rinsed three times with PBS. Then, the cells were fixed in 4% paraformaldehyde for 15 minutes and permeabilized by 0.5% Triton X‐100 (Beyotime, Jiangsu, China) for 10 minutes at room temperature. After blocking for 10 minutes using 1% bovine serum albumin, the primary antibody against vimentin was incubated for 2 hours, followed by Cy3‐labelled secondary antibody (1:100 dilution; Boster Biological Engineering, Wuhan, China) incubation for 1 hour. Nuclei were counterstained with DAPI (KeyGEN Biotech, Nanjing, China) for 5 minutes. The remodelling cells were captured and measured under an Olympus microscope.

### 2.8 Western blot analysis

Total proteins in cultured chondrocytes were extracted using RIPA buffer containing protease and phosphatase inhibitors. Bicinchoninic acid (BCA) reagents (ThermoFisher Scientific, 23225, Waltham, MA, USA) were used to measure protein concentrations. Total proteins were loaded onto 10% sodium dodecyl sulphate‐polyacrylamide gel electrophoresis (SDS‐PAGE). Following electrophoretic separation, the samples were transferred onto polyvinylidene difluoride membranes and then blocked in 5% non‐fat milk. The primary antibodies of cleaved caspase‐3, Bcl‐2, Bax, iNOS, p‐ATF2, ATF2, p‐JNK, JNK, p‐p38 MAPK, p38 MAPK and GAPDH were incubated overnight at 4°C. The respective peroxidase‐conjugated secondary antibodies were added and incubated for 1 hour at room temperature. Finally, the Western blots were visualized using the Odyssey infrared imaging system (LI‐COR, NE, USA) after reaction with a high‐sensitivity chemiluminescence reagent (Amersham Biosciences, Piscataway, NJ, USA).

### 2.9 Animal model and LIG treatment

The animal experiments were permitted by the Animal Care and Use Committee of Medical School, Wuhan University. Male SD rats (200‐250 g body weight) were purchased from the Center for Animal Experiment/ABSL‐III Laboratory of Wuhan University. Rats were randomly divided into five groups (n* *=* *5): sham‐operated, LIG (High‐dose), OA‐induced, OA + LIG (Low‐dose) and OA + LIG (High‐dose) groups. The animal OA model was established using anterior cruciate ligament transection together with medial menisci resection (ACLT + MMx) on the right knee joint.^19^ The rats were located in an electric circling cage for 30 minutes each day at 1 week after surgery as previously described.^20^ Four weeks after surgery, intra‐articular injection in low‐ and high‐dose LIG groups was performed with 30 μL of 75 μmol/L and 150 μmol/L LIG. Meanwhile, the sham‐operated and OA‐induced groups received 30 μL of PBS injection. The whole experimental rats were killed at the 10th week after surgery.

### 2.10 Micro‐CT evaluation

The effects of LIG on micro‐architectural degeneration in the rat OA model were evaluated using micro‐CT equipment (Scam Xmate‐E090, Comscantechno Co., Kanagawa, Japan). Appropriate images were acquired with a resolution ratio of 4000 × 2672 pixels and an isotropic voxel extent of 9 μm. The quantitative assessment was calculated from micro‐tomographic data. The following two indexes were measured: (a) bone mineral density (BMD, g/cm^3^) and (b) bone volume fraction (BV/TV, %).

### 2.11 Histopathologic analysis

Specimens were immediately fixed in 4% paraformaldehyde for 24 hours, placed in 10% EDTA decalcification solution for 4 weeks and subsequently embedded in paraffin wax. The 5 μm serial sagittal sections covering whole joints were stained with Haematoxylin and eosin staining and toluidine blue‐O staining. The level of cartilage degeneration was evaluated by two observers in a blinded manner using a modified Mankin scoring system. Meanwhile, the safranin‐O‐Fast green staining was performed to value cartilage proteoglycan.

### 2.12 TUNEL analysis

TUNEL assays were conducted using an in situ cell death detection kit in accordance with the manufacturer's instructions. Briefly, knee joints in serial sagittal sections were pretreated with 20 μg/mL proteinase K (Dako, Glostrup, Denmark) for 15 minutes, followed by the inhibition of 3% hydrogen peroxide for 5 minutes. The terminal deoxynucleotidyl transferase was applied directly to the tissue sections for 1 hour. Sections were then incubated with anti‐digoxigenin‐peroxidase antibody for 30 minutes, labelled by diaminobenzidine and then stained with haematoxylin. The apoptotic chondrocyte ratio was measured using five casually selected high‐power fields (400 × ) from five groups.

### 2.13 Immunohistochemical analysis

The protein expression levels of p‐ATF2, p‐JNK and p‐p38 MAPK were detected on knee joint cartilage using immunohistochemical analyses. Immunohistochemical staining was carried out using the streptavidin‐peroxidase method. The tissue sections were incubated with primary antibodies at 4°C overnight, followed by the biotinylated secondary antibody. The positive expression of p‐ATF2, p‐JNK and p‐p38 MAPK appeared brown. The number of immunoreactive cells in sections was analysed using Image‐Pro Plus 6.0 analysis software (Media Cybernetics, Inc., Silver Spring, MD, USA).

### 2.14 Statistical analysis

Data were presented as the mean ± standard error of the mean (SEM) for each group. Within‐group differences were performed by one‐way analysis of variance and Student's *t* test with SPSS 16.0 software. Statistical significance was considered at the 0.05 level of probability (*P *<* *0.05). Data were graphically accomplished using GraphPad Prism software, version 5.0 (San Diego, CA, USA).

## 3 RESULTS

### 3.1 LIG promotes cell viability and inhibits SNP‐induced chondrocyte apoptosis

The cell viability at various concentrations of LIG treatment for 12 and 24 hours was examined by the CCK‐8 assay on rat articular chondrocytes. Figure 1B shows that LIG at 25‐100 μmol/L exerted no cytotoxic effect on rat chondrocytes.

To explore the effect of LIG on SNP‐induced chondrocyte apoptosis, we measured the cell apoptotic ratios through flow cytometry. In Figure 1C and D, the percentage of apoptotic chondrocytes stimulated with 0.75 mmol/L SNP was 55.5% ± 2.3%, which was significantly higher than that of the control group (***P <* *0.001). The percentage of apoptotic chondrocytes decreased when the concentration of LIG increased from 25 to 50 μmol/L, and the apoptotic rates were 43.3% ± 2.7% and 34.2% ± 1.0% respectively. The pretreatment with LIG plus SB203580 (10 μmol/L) or SP600125 (10 μmol/L) significantly enhanced the BBR‐mediated decrease in the cell apoptotic rates in the SNP‐stimulated rat chondrocytes (^&&^ *P *<* *0.01 and ^&&&^ *P *<* *0.001).

### 3.2 LIG attenuates nucleic morphological changes and cytoskeletal remodelling and increases mitochondrial membrane potential in SNP‐stimulated rat chondrocytes

**Figure 2:**
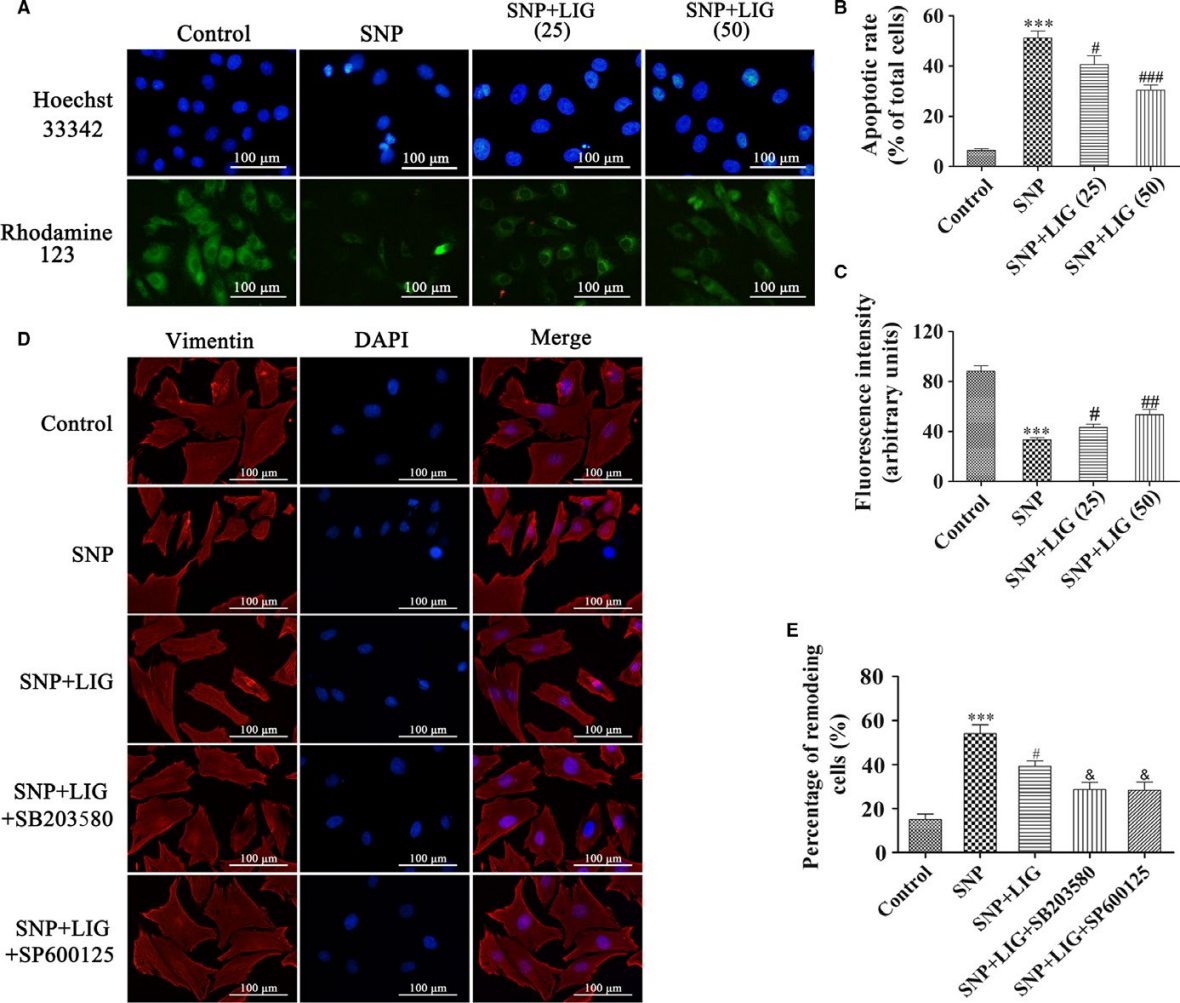
Effects of ligustilide (LIG) on nuclear morphology, mitochondrial membrane potential and cytoskeletal remodelling in sodium nitroprusside (SNP)‐stimulated chondrocytes. (A, B, C) Hoechst 33342 and Rhodamine‐123 staining of chondrocytes exposed to LIG (25 and 50 μmol/L) for 2 h before 0.75 mmol/L SNP co‐treatment for 24 h. The levels of chondrocyte nucleic morphologic changes and intracellular Rhodamine‐123 fluorescence were evaluated. (D, E) Fluorescent images with Vimentin‐Tracker red of chondrocytes pre‐incubated with LIG (50 μmol/L) in the presence and absence of the JNK inhibitor SP600125 (10 μmol/L) and the p38 mitogen‐activated protein kinase (MAPK) inhibitor SB203580 (10 μmol/L) for 2 h before 0.75 mmol/L SNP co‐treatment for 24 h, and the cell nuclei were stained with DAPI. The percentage of remodelling cells was measured. Each column represented mean ± SEM (n = 5). ***P < 0.001 vs the control group; ^#^ *P *< 0.05, ^##^ *P *< 0.01 and ^###^ *P *< 0.001 vs the SNP group; ^&^ *P *< 0.01 vs the SNP + LIG (50 μmol/L) group

We initially surveyed the changes in cell nucleic morphology and mitochondrial membrane potential by Hoechst 33342 nuclear staining and Rhodamine‐123 staining. In Figure 2A‐C, chondrocyte nuclei were round and stained homogeneously in the control group. In the SNP group, approximately 51.2% ± 6.1% of the chondrocytes exhibited compressed and uneven nuclei. When the concentration of LIG increased from 25 to 50 μmol/L, the ratio of chondrocyte nuclear condensation and disintegration decreased significantly compared with that of the SNP group (^#^ *P *<* *0.05 and ^###^ *P *<* *0.001). The mitochondrial membrane potential in the SNP group significantly decreased compared with that in the control group (***P <* *0.001), and this observation could be reversed by 25 to 50 μmol/L LIG (^#^ *P *<* *0.05 and ^##^ *P *<* *0.01).

The vimentin expression in the chondrocytes was measured by immunofluorescence staining to determine the effect of LIG on SNP‐stimulated cytoskeletal remodelling. In Figure 2D and E, the regular vimentin filaments and the uniformly intermediate filament cytoskeleton were observed in the control group. SNP markedly shortened vimentin filaments and stimulated cell shrinkage, which could be prevented by 50 μmol/L LIG treatment. The pretreatment with LIG plus SB203580 (10 μmol/L) or SP600125 (10 μmol/L) significantly enhanced the BBR‐mediated suppression in cytoskeletal remodelling in SNP‐stimulated rat chondrocytes (^&^ *P *<* *0.05). These results indicated that LIG significantly suppressed chondrocyte cytoskeletal remodelling, maintained the nuclear morphology and increased the mitochondrial membrane potential.

### 3.3 LIG attenuates SNP‐stimulated chondrocyte apoptosis by suppressing JNK and p38 MAPK activations

**Figure 3:**
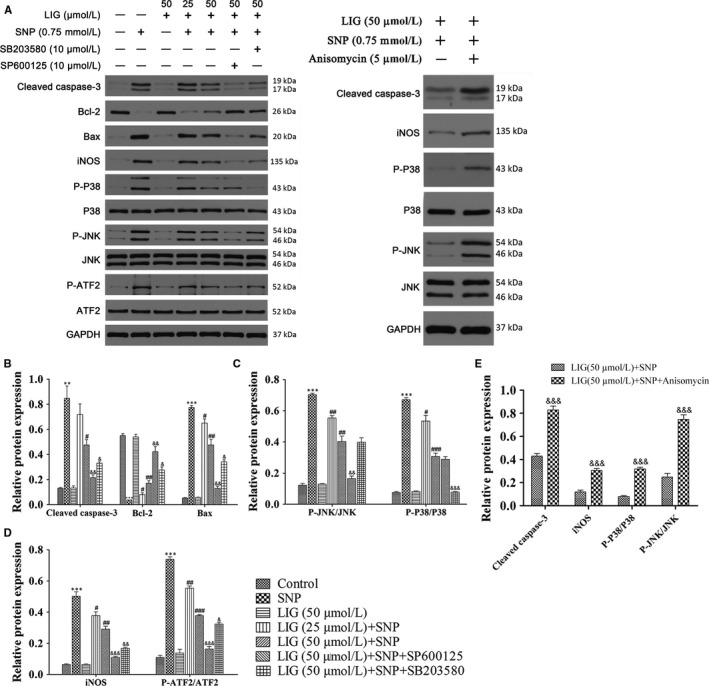
JNK and p38 mitogen‐activated protein kinase (MAPK) signalling pathways are involved in the anti‐apoptotic effect of ligustilide (LIG) on sodium nitroprusside (SNP)‐stimulated rat chondrocytes. Chondrocytes were pre‐incubated with LIG (25 and 50 μmol/L) in the presence and absence of the JNK inhibitor SP600125 (10 μmol/L), the p38 MAPK inhibitor SB203580 (10 μmol/L) or the agonist anisomycin (5 μmol/L) of both pathways for 2 h before 0.75 mmol/L SNP co‐treatment for 24 h. A, The protein expression levels of cleaved caspase‐3, Bcl‐2, Bax, iNOS, p‐p38 MAPK, p38 MAPK, p‐JNK, JNK, p‐activating transcription factor 2 (ATF2) and p‐ATF2 were determined using Western blot; glyceraldehyde‐3‐phosphate dehydrogenase (GAPDH) was used as the loading control. B, The ratios of cleaved caspase‐3, Bcl‐2 and Bax to GAPDH were analysed. C, The ratios of p‐JNK/JNK and p‐p38/p38 were analysed. D, The ratios of inducible nitric oxide synthase (iNOS)/GAPDH and p‐ATF2/ATF2 were examined. E, The ratios of cleaved caspase‐3 and iNOS to GAPDH, p‐JNK/JNK and p‐p38/p38 were examined. Each column represented mean ± SEM (n = 3). **P < 0.01 and ***P < 0.001 vs the control group; ^#^ *P *< 0.05, ^##^ *P *< 0.01 and ^###^ *P *< 0.001 vs the SNP group; ^&^ *P *< 0.05, ^&&^ *P *< 0.01 and ^&&&^ *P *< 0.001 vs the SNP + LIG (50 μmol/L) group

The expression levels of the cleaved caspase‐3, Bcl‐2, Bax and iNOS were measured through Western blot to investigate the mechanism by which LIG suppressed chondrocyte apoptosis. In Figure 3, the expression levels of cleaved caspase‐3, Bax and iNOS significantly increased and that of Bcl‐2 significantly decreased in the SNP‐stimulated chondrocytes compared with those of the control group (**P <* *0.01 and ***P <* *0.001). These trends were reversed by 25 to 50 μmol/L LIG. The pretreatment with LIG plus SB203580 (10 μmol/L) or SP600125 (10 μmol/L) significantly enhanced the LIG‐mediated increase in the Bcl‐2 expression and decrease in the cleaved caspase‐3, Bax and iNOS expression. When the agonist anisomycin (5 μmol/L) of p38 MAPK and JNK pathways was added to the treatment, the LIG‐mediated decrease in the cleaved caspase‐3 and iNOS expression was reversed. The total and phosphorylation levels of ATF2, JNK and p38 MAPK in the different groups were assessed. In Figure 3C‐E, the expression levels of p‐ATF2, p‐JNK and p‐p38 MAPK were higher in the 0.75 mmol/L SNP‐stimulated chondrocytes than in the control group, whereas the pretreatment with LIG at 25‐50 μmol/L blocked the SNP‐stimulated up‐regulation of p‐ATF2, p‐JNK and p‐p38 MAPK. The p38 MAPK inhibitor SB203580 (10 μmol/L) extensively diminished the expression of p‐ATF2 and p‐p38 MAPK, the JNK inhibitor SP600125 (10 μmol/L) significantly decreased the expression of p‐ATF2 and p‐JNK, and the agonist anisomycin (5 μmol/L) of both pathways extensively increased the expression of p‐p38 MAPK and p‐JNK, compared with the LIG‐mediated regulation. The total levels of ATF2, JNK and p38 MAPK in the different groups displayed no obvious changes. These results showed that LIG performed an anti‐apoptotic function and suppressed JNK and p38 MAPK activation in SNP‐stimulated chondrocytes.

### 3.4 Intra‐articular injection of LIG delays the progression of cartilage degeneration in the ACLT + MMx rat OA model

**Figure 4:**
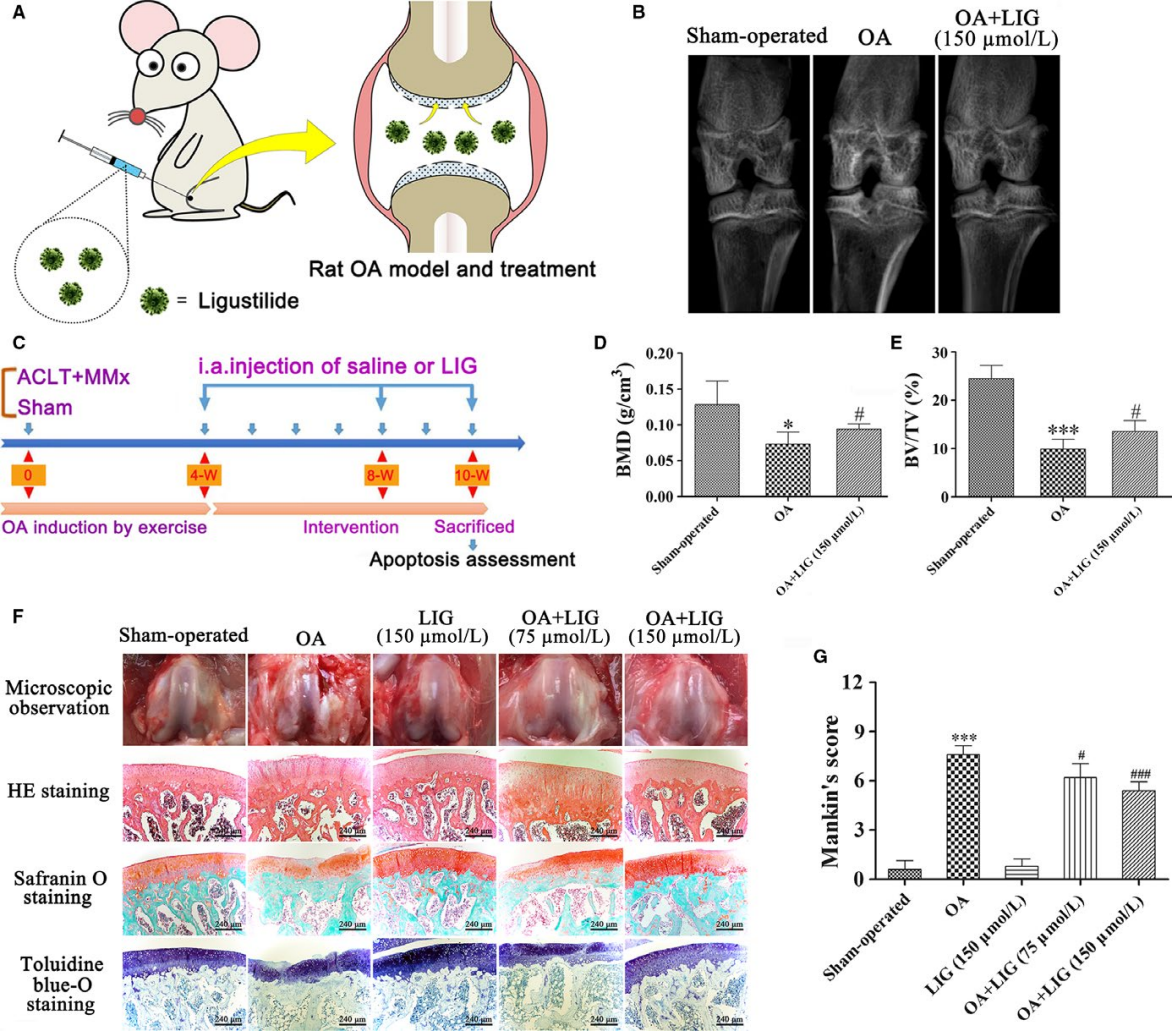
Schematic depicting the time of treatment, the follow‐up period of the animals and the effects of ligustilide (LIG) on cartilage degradation in rat articular cartilage 10 weeks after anterior cruciate ligament transection together with medial menisci resection (ACLT + MMx). (A, C) ACLT + MMx rats were placed in an electric rotating cage for 30 min per day to induce the osteoarthritis (OA) model from the 1st week after surgery. Low‐ and high‐dose LIG treatment animals were injected intra‐articularly with 30 μL of 75 and 150 μmol/L LIG 4 weeks after surgery. The sham‐operated and OA‐induced animals received an injection of 30 μL of PBS. The protective effects of LIG on OA progression in vivo were assessed 10 weeks after surgery. (B) Representative micro‐computed tomography two‐dimensional reconstructions of tibial and femoral subchondral bones in the rats after ACLT + MMx surgery or sham operation. (D, E) Bone mineral density (BMD) and bone volume fraction (BV/TV) were measured in the subchondral bones of the knee joint of vehicle‐treated and LIG‐treated rats 10 weeks after ACLT + MMx surgery or sham operation. (F) Gross morphological and histological analyses of rat articular cartilage by H&E staining, Safranin O staining and toluidine blue‐O staining (original magnification 400 ×) in each group. (G) Overall Mankin's histological score was assessed in five groups 10 weeks after ACLT + MMx surgery or sham operation. Each column represented mean ± SEM (*n* = 5). *P < 0.05 and ***P < 0.001 vs the sham‐operated group; ^#^ *P *< 0.05 and ^###^ *P *< 0.001 vs the OA‐induced group

We assessed the potential protective effects of LIG on the OA progression in vivo. In Figure 4B,D and E, micro‐CT was performed to assess the joint space, osteophyte formation and calcification changes in the joint articular cartilage. BMD and BV/TV were down‐regulated in the OA‐induced group compared with those in the sham‐operated group (*P <* *0.05 and ***P <* *0.001). After LIG (150 μmol/L) was intra‐articularly injected, the BMD and BV/TV measurements were significantly higher than those of the OA‐induced group (^#^ *P *<* *0.05). Macroscopic observations are presented in Figure 4F. The cartilage of the femoral condyles was smooth and did not have osteophytes in the sham‐operated group, whereas the cartilage surface was rough and had serious erosion in the OA‐induced group. After LIG was intra‐articularly injected at 75‐150 μmol/L, the severity of cartilage degradation was reversed to varying degrees.

Histological analysis was performed by HE staining, Safranin O staining and toluidine blue‐O staining of the articular cartilage (Figure 4F). In the sham‐operated and LIG groups, an even surface and regular chondrocytes were detected in the cartilage, whereas disordered chondrocyte clusters and an uneven cartilage surface were observed in the OA‐induced group. No significant lesions were found, a slight erosion was identified on the cartilage surface, and the chondrocytes re‐established a well‐organized pattern in the OA + LIG groups in a dosage‐dependent manner. According to the modified Mankin scores (Figure 4G), the severity degrees of cartilage degradation in the OA + LIG (75 and 150 μmol/L) groups were lower than that in the OA‐induced group (^#^ *P *<* *0.05 and ^###^ *P *<* *0.001).

### 3.5 Inhibition of JNK and p38 MAPK signalling pathways is regulated by LIG in the ACLT + MMx rat OA model

**Figure 5:**
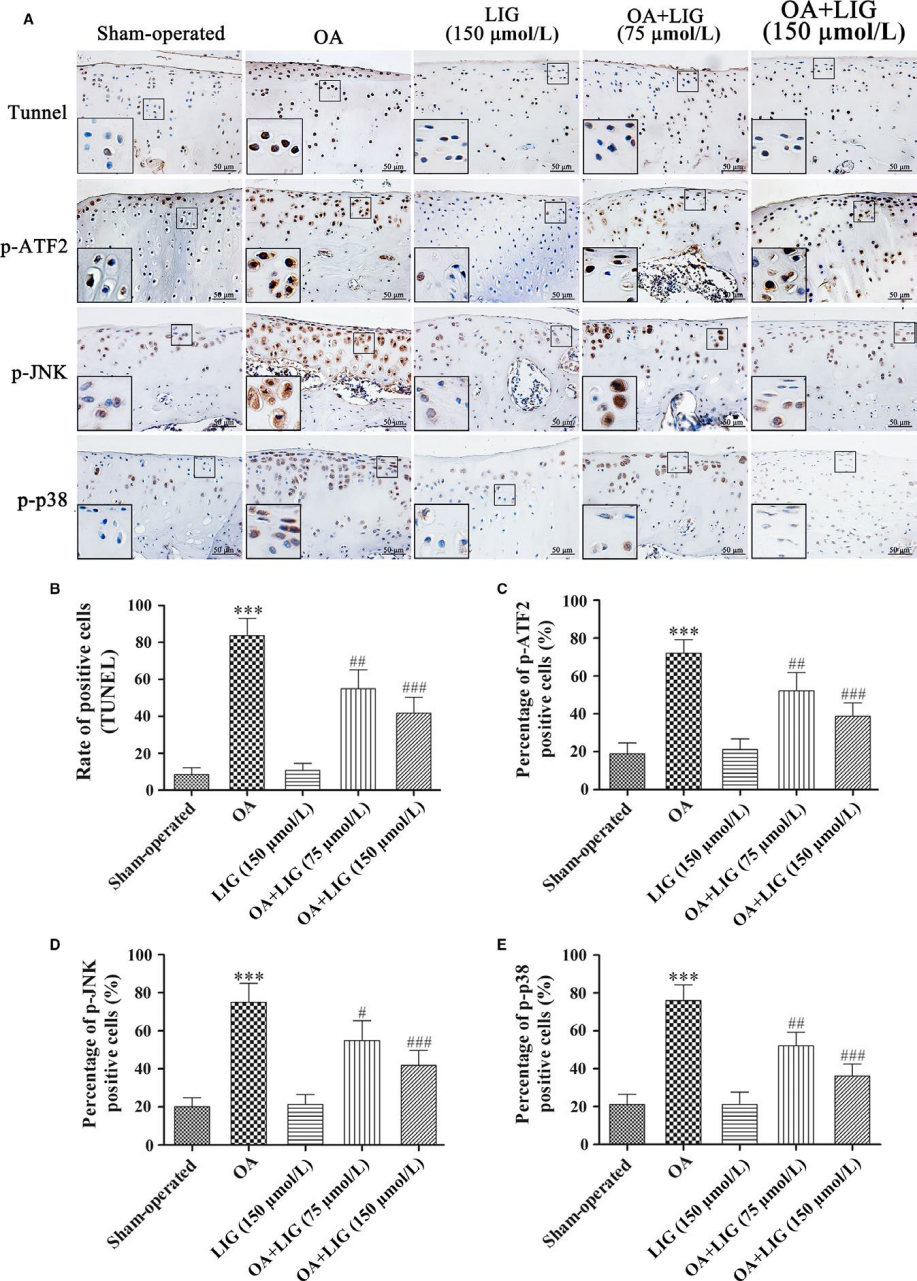
Effects of ligustilide (LIG) on the JNK and p38 mitogen‐activated protein kinase (MAPK) pathways in cartilage chondrocytes in vivo. (A) Apoptotic chondrocytes in the rat articular cartilage were detected by TUNEL staining (original magnification 400 ×). Immunohistochemical analyses of p‐activating transcription factor 2 (ATF2), p‐JNK and p‐p38 MAPK expression were determined in each group (original magnification 400 × and zoom‐in areas magnification 800 ×) 10 weeks after anterior cruciate ligament transection together with medial menisci resection (ACLT + MMx) surgery or sham operation. (B) The percentage of apoptotic chondrocytes in articular cartilage was calculated. (C‐E) The ratios of immunoreactive positive cells of p‐ATF2, p‐JNK and p‐p38 MAPK were analysed. Each column represented mean ± SEM (n = 5). ***P < 0.001 vs the sham‐operated group; ^#^ *P *< 0.05, ^##^ *P *< 0.01 and ^###^ *P *< 0.001 vs the osteoarthritis‐induced group

The biological effects of LIG on chondrocyte apoptosis were explored. The extent of cell death was assessed by TUNEL staining. TUNEL‐positive chondrocytes were intensely expressed in the OA‐induced group, whereas a few chondrocytes were expressed in the sham‐operated group (Figure 5A). The amount of TUNEL‐positive chondrocytes was strongly decreased by the LIG treatment in a dosage‐dependent manner. The ratio of TUNEL‐positive chondrocytes in the five groups is presented in Figure 5B, and significant differences were detected between the OA‐induced and OA + LIG groups (^##^ *P *<* *0.01 and ^###^ *P *<* *0.001).

The expression levels of p‐ATF2 and the inhibition of JNK and p38 MAPK signalling pathways in the articular cartilage were detected through immunohistochemical staining. In Figure 5A,C‐E, the percentages of p‐ATF2, p‐JNK and p‐p38‐positive chondrocytes in the OA‐induced group were substantially higher than those in the sham‐operated group (***P <* *0.001). The percentages of p‐ATF2, p‐JNK and p‐p38 were significantly decreased by the 75‐150 μmol/L LIG treatment compared with those of the OA‐induced group (^#^ *P *<* *0.05, ^##^ *P *<* *0.01 and ^###^ *P *<* *0.001).

## 4 DISCUSSION

In this study, we explored the effects of LIG on SNP‐stimulated rat chondrocyte apoptosis and articular cartilage in the ACLT + MMx rat OA model and clarified the underlying mechanism involving the JNK and p38 MAPK signalling pathways. Our data provided molecular evidence that LIG decreased SNP‐stimulated chondrocyte apoptosis and elicited protective effects on articular cartilage by suppressing JNK and p38 MAPK activities.

Osteoarthritis is caused by various factors, such as biomechanical forces, metabolic distress, cytokine occurrence, genetic heritage, cartilage condition, chondrocyte apoptosis and free radical generation.^21^ Chondrocyte apoptosis is generally considered a vital pathological feature of OA. Weakening the course of chondrocyte apoptosis can ameliorate the progress of joint cartilage degeneration. LIG, a quinone extracted from the roots of *Plumbago* plants, has been applied widely to treat various diseases because of its anti‐apoptotic property. For example, the anti‐apoptotic property of LIG may reduce ischaemia/reperfusion‐induced increase in brain iron.^22^ LIG can inhibit the hypertrophy of cardiomyocytes stimulated by Ang II, which may be attributed to the ability of LIG to suppress cardiomyocyte apoptosis.^23^ LIG can protect C2C12 cells from TNF‐α‐induced apoptosis during differentiation by inhibiting apoptosis and inducing cell proliferation.^24^ Given its anti‐apoptotic effect on neurons, LIG can be developed as an effective drug for the prevention of vascular dementia.^25^ However, the anti‐apoptotic effect of LIG on OA chondrocytes is largely unknown. As an important signalling molecule, NO plays a vital role in several physiological and biochemical processes, including blood pressure control, nervous system transmission, immune response and cell apoptosis. Clinical and basic studies have demonstrated that SNP is a fast vasodilator and exogenous NO donor, which can release a NO radical in a solution and induce the biological effect of apoptosis. Our study assessed the role of LIG in apoptotic chondrocytes and examined whether LIG decreased SNP‐stimulated chondrocyte apoptosis. As a crucial enzyme related to apoptosis, caspase‐3 plays a vital role in chondrocyte apoptosis. The heterodimer formed by Bcl‐2 protein and Bax at the early stage of apoptosis is considered an apoptosis promoter, which controls cell death. The expression of Bcl‐2 and Bax and the relationship between these two proteins may result in the induction of cell apoptosis. In this study, Bcl‐2, Bax and cleaved caspase‐3 were selected as the measurable indicators of cell apoptosis. Our findings suggested that LIG inhibited SNP‐stimulated apoptosis in chondrocytes by shifting the balance of Bcl‐2 and Bax and attenuating the activation of cleaved caspase‐3. Chondrocyte homeostasis needs an integral cytoskeleton and extracellular matrix synthesis, and the disorder of the vimentin system may accelerate cartilage degradation.^26^ Our immunofluorescent analysis suggested that LIG could reverse the SNP‐induced vimentin cytoskeletal remodelling. Mitochondrial dysfunctions containing the loss of mitochondrial membrane potential and the decrease in adenosine triphosphate production are typical hallmarks of apoptosis.^27^ This process was also confirmed by Hoechst 33342 staining and flow cytometry analysis in SNP‐stimulated chondrocytes in vitro. There is mitochondrial functional deletion in OA chondrocytes, and mitochondrial function deletion may be prior to the process of apoptosis.^28^ Cell surface receptors and mitochondrial membrane permeability are activated during apoptosis, and chromosomal DNA structure changes, thereby enabling the efficient binding of dyes to DNA. Our in vitro study confirmed that the overproduction of cleaved caspase‐3, Bcl‐2, Bax and iNOS after SNP stimulation was reversed by LIG at protein levels, and LIG exhibited anti‐apoptotic and protective effects on OA chondrocytes as demonstrated by the inhibition of mitochondrial membrane permeability and the stabilization of the chromosomal DNA structure in the SNP‐stimulated rat chondrocytes.

The responses of the JNK and p38 MAPK pathways to LIG treatment in the SNP‐stimulated rat chondrocytes were studied to further explore the underlying mechanisms and signalling pathways related to the anti‐apoptotic activity of LIG. Our results showed that SNP induced the phosphorylation of JNK and p38 MAPK, whereas the pretreatment with LIG restrained the activation of JNK and p38 MAPK activities. Existing studies have indicated the vital role of MAPK pathways in mechanical stress or heat and NO‐stimulated chondrocyte apoptosis.^29^, ^30^ MAPK is an important signal transduction pathway that regulates numerous physiological processes and plays an important role in the initiation of cell damage. The activation of MAPK results in the phosphorylation of JNK and p38 MAPK, triggers transcription factors and stimulates cell apoptosis.^31^ In addition, p38 MAPK is a vital signalling pathway that activates Bax subsequent to its translocation to the mitochondria.^32^ The stimulation of iNOS/NO influences cartilage homeostasis, which leads to the apoptosis of chondrocytes and the suppression of matrix synthesis.^33^ A decrease in iNOS is related to the amelioration of cartilage breakdown and chondrocyte apoptosis, which has been verified to relieve pain and inflammation related to OA symptoms.^34^ JNK and p38 MAPK pathways trigger the transcription factor ATF2 that stimulates the iNOS promoter activity.^35^ Our data showed that LIG prevented the SNP‐induced activation of JNK and p38 MAPK that consequently hindered the activation of ATF2 resulting in declined iNOS production. Interestingly, the pretreatment with SP600125 or SB203580 significantly enhanced and anisomycin offset the LIG‐induced effects, including the inhibition of ATF2, JNK and p38 MAPK phosphorylation, the down‐regulation of iNOS release and cleaved caspase‐3 activity, as well as the anti‐apoptotic effect. These results indicated that the anti‐apoptotic and protective effects of LIG on OA chondrocytes occurred by regulating the JNK and p38 MAPK pathways, which might be the potential molecular mechanisms of the protective effects of LIG.

Osteoarthritis is a degenerative chondropathy that involves a combination of cartilage degradation, chondrocyte apoptosis and inflammation.^36^ The main consequences of interest in the weighing beneficial value of preclinical OA models generally depend on the anatomical indication of articular cartilage and synovial pathology and the imaging form.^37^ From a clinical perspective, joint function restoration and characteristic pain alleviation are more important than the structural or radiological indication of pathology. Recent progress on non‐destructive imaging modalities, such as magnetic resonance imaging and micro‐CT, has led to the formation of three‐dimensional images of bones and joints of laboratory animals and possibly humans.^38^ Furthermore, the use of micro‐CT has effectively delivered the direct in situ imaging of joint articular cartilage in preclinical OA models and the evaluation of joint spaces, osteophyte construction and calcification on the articular cartilage surface.^39^ In the in vivo study, the OA‐induced group displayed joint space lessening, irregular osteophytes and calcification compared with those of the sham‐operated group. The pathological phenotypes were relieved after LIG was administered as demonstrated by micro‐CT and confirmed by histological analysis. Our data further suggested that LIG decreased articular chondrocyte apoptosis and ameliorated cartilage degeneration in the rat ACLT + MMx OA model. Considerably lower Mankin scores, greater matrix synthesis and thicker cartilage layer were detected in the LIG‐injected groups compared with those in the OA‐induced group. TUNEL assay demonstrated that LIG suppressed cartilage chondrocyte apoptosis in the rat OA model. Immunohistochemical results indicated that the intra‐articular injection of LIG reduced the phosphorylation level of ATF2, JNK and p38 MAPK protein expression in the articular cartilage. Thus, LIG showed a prospective therapeutic capacity for cartilage erosion in vivo.

**Figure 6:**
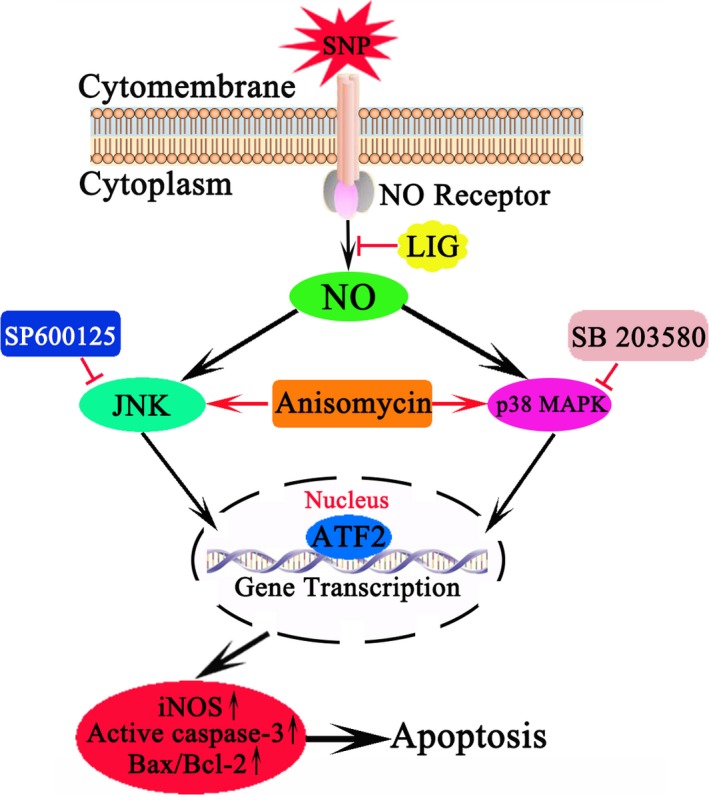
Proposed mechanism of ligustilide (LIG) interference with osteoarthritis chondrocyte apoptosis. LIG inhibited chondrocyte apoptosis and ameliorated cartilage degeneration via the JNK and p38 mitogen‐activated protein kinas (MAPK) signalling pathways, leading to the down‐regulation of inducible nitric oxide synthase (iNOS) and p‐activating transcription factor 2 (ATF2) expression

Based on these concepts, a hypothetical pathological process for OA was presented as follows (Figure 6). A signal reached the chondrocyte surface and triggered the matching receptor, resulting in the production of a following messenger, such as NO, which then stimulated JNK/p38 protein kinase. The manner could be defined as NO → JNK/p38 MAPK → ATF2. Afterwards, ATF2 translocated to the chondrocyte nucleus, where it stimulated and regulated the expression of apoptotic genes, such as cleaved caspase‐3, Bcl‐2, Bax and iNOS. LIG treatment markedly reversed the pathological process and suppressed JNK and p38 MAPK activations.

In summary, our results collectively suggested that LIG inhibited NO‐induced chondrocyte apoptosis and exerted protective effects on articular cartilage by suppressing JNK and p38 MAPK pathways. These findings supported the potential therapeutic value of LIG in OA treatment.

## CONFLICTS OF INTEREST

The authors confirm that there are no conflicts of interest.
